# Adjuvant therapy after resection of colorectal liver metastases: the predictive value of the MSKCC clinical risk score in the era of modern chemotherapy

**DOI:** 10.1186/1471-2407-14-174

**Published:** 2014-03-11

**Authors:** Nuh N Rahbari, Christoph Reissfelder, Henning Schulze-Bergkamen, Dirk Jäger, Markus W Büchler, Jürgen Weitz, Moritz Koch

**Affiliations:** 1 Department of General, Visceral and Transplantation Surgery, University of Heidelberg, Heidelberg, Germany; 2 Department of Medical Oncology, National Center of Tumor Diseases, University of Heidelberg, Heidelberg, Germany; 3 Department of Visceral-, Thoracic and Vascular Surgery University Hospital Carl Gustav Carus, Technical University Dresden, Fetscherstr. 74, D-01307 Dresden, Germany

**Keywords:** Colorectal cancer, FOLFOX, FOLFIRI, 5-FU, Leucovorin, Liver resection

## Abstract

**Background:**

Despite introduction of effective chemotherapy protocols, it has remained uncertain, if patients with colorectal cancer (CRC) liver metastases should receive adjuvant therapy. Clinical or molecular predictors may help to select patients at high risk for disease recurrence and death who obtain a survival advantage by adjuvant chemotherapy.

**Methods:**

A total of 297 patients with potentially curative resection of CRC liver metastases were analyzed. These patients had no neoadjuvant therapy, no extrahepatic disease and negative resection margins. The primary endpoint was overall survival. Patients’ risk status was evaluated using the Memorial Sloan-Kettering Cancer Center clinical risk score (MSKCC-CRS). Multivariable analyses were performed using Cox proportional hazard models.

**Results:**

A total of 137 (43%) patients had a MSKCC-CRS > 2. Adjuvant chemotherapy was administered to 116 (37%) patients. Patients who received adjuvant chemotherapy were of younger age (p = 0.03) with no significant difference in the presence of multiple metastases (p = 0.72) or bilobar metastases (p = 0.08). On multivariate analysis adjuvant chemotherapy was associated with improved survival in the entire cohort (Hazard ratio 0.69; 95% confidence interval 0.69–0.98). It improved survival markedly in high-risk patients with a MSKCC-CRS > 2 (HR 0.40; 95% CI 0.23–0.69), whereas it was of no benefit in patients with a MSKCC-CRS ≤ 2 (HR 0.90; 95% CI 0.57–1.43).

**Conclusions:**

The MSKCC-CRS offers a tool to select patients for adjuvant therapy after resection of CRC liver metastases. Validation in independent patient cohorts is required.

## Background

The liver represents the most frequent metastatic site in patients with colorectal cancer (CRC). At the time of diagnosis some 20–30% of CRC patients present with synchronous liver metastases and a similar proportion of patients will develop metastatic disease to the liver after radical resection of the colorectal primary [1]. At present, complete surgical resection is the primary therapy for patients with CRC liver metastases and in selected cases may enable 5-year survival rates of 30–50% [2,3].

Studies on the treatment of primary CRC have fueled discussions, if adjuvant chemotherapy improves survival after resection of CRC liver metastases [4-6]. The available randomized controlled trials did not evaluate modern chemotherapy protocols and, moreover, lack sufficient power to draw final conclusions [7]. The EORTC Intergroup Trial 40983 showed a significant benefit in progression-free survival in eligible patients who received pre-*and* postoperative therapy with the FOLFOX-4 protocol compared to surgery alone [8]. Disadvantages of neoadjuvant therapy such as increased perioperative morbidity, parenchymal injury and reduced treatment options in case of disease recurrence need to be considered and possibly outweigh the observed benefit in progression-free survival. The long-term results of the EORTC Intergroup Trial 40983 indeed did not show a significant improvement in five-year overall survival in the perioperative chemotherapy group [9]. The available clinical data therefore clearly demonstrate the need for strategies to tailor adjuvant therapy to patients who are likely to obtain a marked therapy-induced benefit in long-term outcome after potentially curative resection of CRC liver metastases.

It was therefore the aim of the present study to evaluate, if patients’ clinical risk profile using the Memorial Sloan-Kettering Cancer Center clinical risk score (MSKCC-CRS) may serve as a tool to predict the efficacy of adjuvant chemotherapy after resection of colorectal liver metastases [2]. This clinical score consists of five criteria (node-positive primary, disease-free interval < 12 months, >1 tumor, tumor size **>** 5 cm, CEA **>**200 ng/ml) and has repeatedly shown adequate prognostic stratification of patients undergoing resection for CRC liver metastases [3,10-12].

## Patients and methods

### Study population

Patients were identified from a prospective database maintained at the Department of General, Visceral and Transplantation Surgery, University of Heidelberg. CRC patients who underwent surgical resection for histologically proven liver metastases between October 2001 and June 2009 and received adjuvant chemotherapy or no adjuvant therapy after potentially curative resection were eligible for the analyses. We excluded patients who had received neoadjuvant therapy prior to hepatic resection, patients who already had previous resection of CRC metastases, patients with extrahepatic disease and those with positive resection margins. Furthermore, patients who received targeted therapy in the adjuvant setting were excluded. Potentially curative surgery was defined as complete resection of all liver metastases, regardless of size, number, distribution, or width of (negative) resection margin and might have been completed by concomitant local ablation of small lesions (< 3 cm in diameter). Tumor stage was classified according to the seventh edition of the TNM classification of the UICC (International Union Against Cancer) [13]. The study was approved by the independent ethics committee of the University of Heidelberg.

### Patient treatment and follow-up

Patients were treated as described previously [3,14-16]. Preoperative work-up included a physical examination, serum CEA levels and a computed tomography (CT) scan of the abdomen and chest. Furthermore, a colonoscopy and pelvic magnetic resonance imaging (MRI) was required in patients with colon and rectal primaries, respectively. Patients with significant medical comorbidities were referred for an extensive cardiopulmonary evaluation. Intraoperative ultrasound of the liver was performed in all patients. Liver resection was carried out under low central venous pressure using a the clamp-crushing technique, ultrasonic dissector or stapler transection [17]. Hepatic inflow control (i.e. portal triad clamping) was not used regularly [18]. After hepatic resection follow-up visits were performed at regular intervals at the outpatient clinics of the Department of Surgery, University of Heidelberg and the National Center for Tumor Diseases (NCT) Heidelberg. Patients were followed with serum CEA measurement, abdominal ultrasound and chest X-ray every three months for the first two years and every six months thereafter. A CT of the chest and abdomen was performed initially after three months and then every six months for two years. In the absence of recurrent disease CT scans were performed annually thereafter.

The decision for adjuvant therapy as well as the chemotherapy protocol was made within a multidisciplinary setting. While the decision for adjuvant chemotherapy was made on an individual basis for each patient, the following factors were taking into account: clinicopathologic factors (i.e. disease-free interval, extent of disease, organ function, recovery from surgery, etc.), extent and tolerance of chemotherapy pretreatment and patients’ preference.

Adjuvant chemotherapy was initiated within 4 to 6 weeks after surgery. Most of the patients selected for a 5-FU/LV-regimen were treated with LV at 500 mg/m^2^ IV over 2 h plus a 24-hour continuous 5-FU infusion at 2600 mg/m2 on day 1, 8, 15, 22, 29, 36, repeated on day 50, for 3 cycles [19]. In a few patients, alternative weekly infusional 5-FU/LV-regimens were applied. Patients selected for an oxaliplatin-based regimen received FOLFOX4 (oxaliplatin at 85 mg/m2 IV over 2 h on day 1, plus leucovorin (LV) at 200 mg/m2 IV over 2 h on day 1 and 2, plus 5-fluorouracil (5-FU) at 400 mg/m2 IV bolus on day 1 and 2, plus a 22-hour continuous 5-FU infusion at 600 mg/m2 for 2 consecutive days, every two weeks [20]. The FOLFIRI regimen was administrated as following: Irinotecan at 180 mg/m2 IV over 1 h on day 1, plus LV at 400 mg/m2 IV over 2 h on day 1, plus 5-FU at 400 mg/m2 IV bolus on day 1, plus a 46-hour continuous 5-FU infusion at 2400 mg/m2, every two weeks [21]. Alternative FOLFOX or FOLFIRI regimens were applied in selected patients who received adjuvant therapy at external institutions.

### Assessment of patients’ risk status

Patients’ risk status was evaluated preoperatively using the MSKCC-CRS [2], as its prognostic value has been confirmed in multiple analyses from various institutions [3,10,11]. This score uses the following five prognostic parameters: size of the largest metastasis > 5 cm, node-positive primary tumor, multiple metastases, preoperative CEA level > 200 ng/ml and disease-free interval from the primary to the diagnosis of liver metastasis < 12 months. Based on the number of criteria met patients are classified into six different risk groups (MSKCC-CRS 0–5).

### Statistical analyses

Continuous data were reported as median (range) and categorical data were expressed as absolute and relative frequencies. Continuous and categorical data were compared with *Student’s t-test, Wilcoxon test* and *Pearson’s χ*^*2*^-test, respectively. The primary endpoint was overall survival. Patients who were lost to follow-up were censored at the date of last contact, as were patients who were alive at the time of the last follow-up visit. Cox proportional hazards regression models were used for multivariate analyses and included known prognosticators in patients with colorectal liver metastases. All p values were two-sided. A p-value < 0.05 was considered to indicate statistical significance. All analyses were done using SPSS® software version 17 (SPSS, Chicago, Illinois, USA) and JMP program version 7 (SAS Institute Inc., Cary, NC, USA).

## Results

A total of 386 patients who underwent resection of colorectal liver metastases during the study period of eight years were identified from the database. After exclusion of patients who had received neoadjuvant therapy, patients with recurrent liver metastases or extrahepatic disease and those with positive resection margins a total of 297 patients with a potentially curative resection of CRC liver metastases remained eligible for final analyses (Table 1). There were 199 (67%) men and 98 (33%) women with a median age of 64 (30–88) years. Of these, 125 (42%) patients had synchronous metastases. The primary tumor was located in the colon and in the rectum in 166 (56%) and 131 (44%) patients, respectively. There were 137 (46%) patients with multiple metastases and 110 (37%) patients with a bilobar distribution of metastatic lesions. A major resection (i.e. resection of > 2 anatomic segments) was carried out in 172 (58%) patients and was performed similarly in patients with a MSKCC-CRS ≤2 (n = 85; 50%) and MSKCC-CRS > 2 (n = 87; 69%). Some 87 (92.5%) patients with stage III disease and 7 (12.5%) patients with stage II disease had received adjuvant chemotherapy after resection of the primary tumor.

**Table 1:** Clinicopathologic characteristics of patients who underwent potentially curative resection for colorectal cancer liver metastases

	**n (%) or median (range)**
**n**	297 (100)
**Gender**	
Male	199 (67)
Female	98 (33)
**Age [years]**	64 (30 – 88)
**Initial stage of disease [UICC]**	
I	22 (7)
II	56 (19)
III	94 (32)
IV	125 (42)
**Site of primary tumor**	
Colon	166 (56)
Rectum	131 (44)
**CEA level [μg/l]**^**1**^	15.1 (0.5 – 7606)
**Time of metastasis**	
Synchronous	125 (42)
Metachronous	172 (58)
**Number of metastases**	
1	160 (54)
> 1	137 (46)
**Size of largest metastasis**	
< 5 cm	143 (48)
≥ 5 cm	154 (52)
**Distribution of metastases**	
Unilobar	186 (63)
Bilobar	111 (37)
**Extent of liver resection**	
Major resection (>2 segments)	172 (58%)
Minor resection (≤2 segments)	125 (42%)
**MSKCC clinical risk score**	
≤ 2	171 (58)
> 2	126 (42)

^1^Prior to resection of colorectal cancer liver metastases.

Fifteen patients received local ablation. In the group of patients with MSKCC-CRS ≤2 three patients with and four patients without adjuvant therapy had local ablations. In the group of patients with a MSKCC-CRS > 2 three patients with and five patients without adjuvant therapy had local ablations. A total of 46 (39.6%) patients with adjuvant chemotherapy after liver resection for colorectal metastases had not received any adjuvant chemotherapy after resection of the primary tumor. However, 54 (29.8%) patients who had no adjuvant chemotherapy after resection of liver metastases, had received adjuvant chemotherapy after resection of the primary tumor. In 25 (8.3%) patients the information on adjuvant chemotherapy was missing. A total of 125 (42%) patients had a MSKCC-CRS > 2. The distribution of patients across the MSKCC-CRS 0 to 5 was 14 (5%), 51 (17%), 106 (36%), 76 (26%), 43 (14%) and 6 (2%) patients. With respect to the further criteria of the MSKCC-CRS 26 (8.7%) patients had a preoperative CEA level > 200 ng/ml, 197 (65.9%) patients a node-positive primary tumor and 178 (59.5%) patients a time interval < 12 months from the diagnosis of CRC to the diagnosis of metastatic disease to the liver.

More than half of the patient cohort did not receive adjuvant therapy (n = 181; 61%) and some 116 (39%) patients were treated with cytotoxic chemotherapy. The median time to the start of adjuvant chemotherapy was 6 weeks (range: 4–8 weeks). The kind of adjuvant chemotherapy was FOLFOX in 62 (53%) patients, FOLFIRI in 16 (14%) patients and 5-FU/Leucovorin in 38 (33%) patients. Patients with a MSKCC-CRS ≤ 2 (n = 111; 62%) were more likely to receive no adjuvant chemotherapy compared to patients with a score > 2 (n = 70; 38%). Administration of 5-FU/Leucovorin was rather balanced between patients with and without a high MSKCC-CRS, whereas patients with a high MSKCC-CRS more frequently received FOLFIRI (Additional file 1: Table S1). The median duration of chemotherapy was 3 months (range: 1.5–6 months). In 9 patients chemotherapy was stopped due to toxicity.

Table 2 presents patients’ clinicopathologic characteristics stratified for the administration of adjuvant therapy. We noticed a significant difference between the two groups regarding patients’ age (p = 0.03). Variables describing the extent of metastatic disease to the liver such as the presence of multiple metastases (p = 0.72), size of metastases ≥ 5 cm (p = 0.06) and presence of bilobar disease (p = 0.08) did not differ significantly among the groups.

**Table 2:** Clinicopathologic characteristics of patients who underwent resection for colorectal cancer liver metastases stratified for the administration of adjuvant therapy

	**No adjuvant CTx**	**Adjuvant CTx**	***P *** **value**
**n**	181 (61)	116 (39)	
**Gender**			0.11
Male	115 (64)	84 (72)	
Female	66 (36)	32 (28)	
**Age [years]**	64 (30 – 88)	63 (33 – 83)	0.03
**Initial stage of disease (UICC)**			0.53
I	23 (7)	9 (8)	
II	37 (21)	17 (15)	
III	56 (32)	36 (31)	
IV	70 (40)	53 (46)	
**Site of primary tumor**			0.14
Colon	95 (52)	71 (61)	
Rectum	86 (47)	45 (39)	
**CEA level [μg/l)**	12.6 (0.6 – 2032)	18.1 (0.5 – 7606)	0.63
**Number of metastases**			0.72
1	99 (55)	61 (53)	
> 1	82 (45)	55 (47)	
**Size of largest metastasis**			0.06
< 5 cm	95 (52)	48 (41)	
≥ 5 cm	86 (48)	68 (59)	
**Distribution of metastases**			0.08
Unilobar	120 (67)	66 (57)	
Bilobar	60 (33)	55 (43)	
**MSKCC clinical risk score**			0.07
≤ 2	111 (61)	60 (52)	
> 2	69 (39)	56 (48)	

Data are presented as n (%) or median (range). CTx, Chemotherapy.

After the date of primary hepatic resection for metastatic disease patients were followed for a median duration of 32 months (3–107 months). A total of 153 (52%) patients died during the follow-up period. Six (2%) patients who were lost to follow-up were censored at the date of last contact.

To evaluate the independent clinical value of adjuvant chemotherapy a multivariate model was built including the kind of adjuvant therapy together with the initial stage of disease, presence of bilobar metastases and the MSKCC-CRS as known prognostic factor in our patient cohort (Table 3). This analysis demonstrated an advantage in overall survival for patients who received adjuvant chemotherapy (Hazard ratio 0.69; 95% confidence interval 0.49–0.98; p = 0.04). Moreover, this multivariate model confirmed a prognostic impact of bilobar metastases (HR 1.68; 95% CI 1.18–2.39; p = 0.004) and a MSKCC-CRS > 2 (HR 1.56; 95% CI 1.04–2.33; p = 0.02).

**Table 3:** Multivariate analysis of factors associated with overall survival in patients who underwent potentially curative resection for colorectal cancer liver metastases

**Variable**	**Comparison**	**Hazard ratio**	**95% CI**	***P *** **Value**
**Age**	-	1.01	0.99 – 1.03	0.20
**Distribution of metastasis**	Bilobar vs. unilobar	1.68	1.18 – 2.39	0.004
**MSKCC Clinical risk score**	3-5 vs. 0-2	1.56	1.04 – 2.33	0.02
**Initial stage of disease (UICC)**				0.29
		Reference		
	2	1.68	0.73 – 3.87	0.21
	3	1.88	0.85 – 4.18	0.11
	4	1.44	0.63 – 3.25	0.38
**Adjuvant chemotherapy**	Adjuvant CTx vs. no adjuvant CTx	0.69	0.49 – 0.98	0.04

CTx, Chemotherapy.

**Figure 1:**
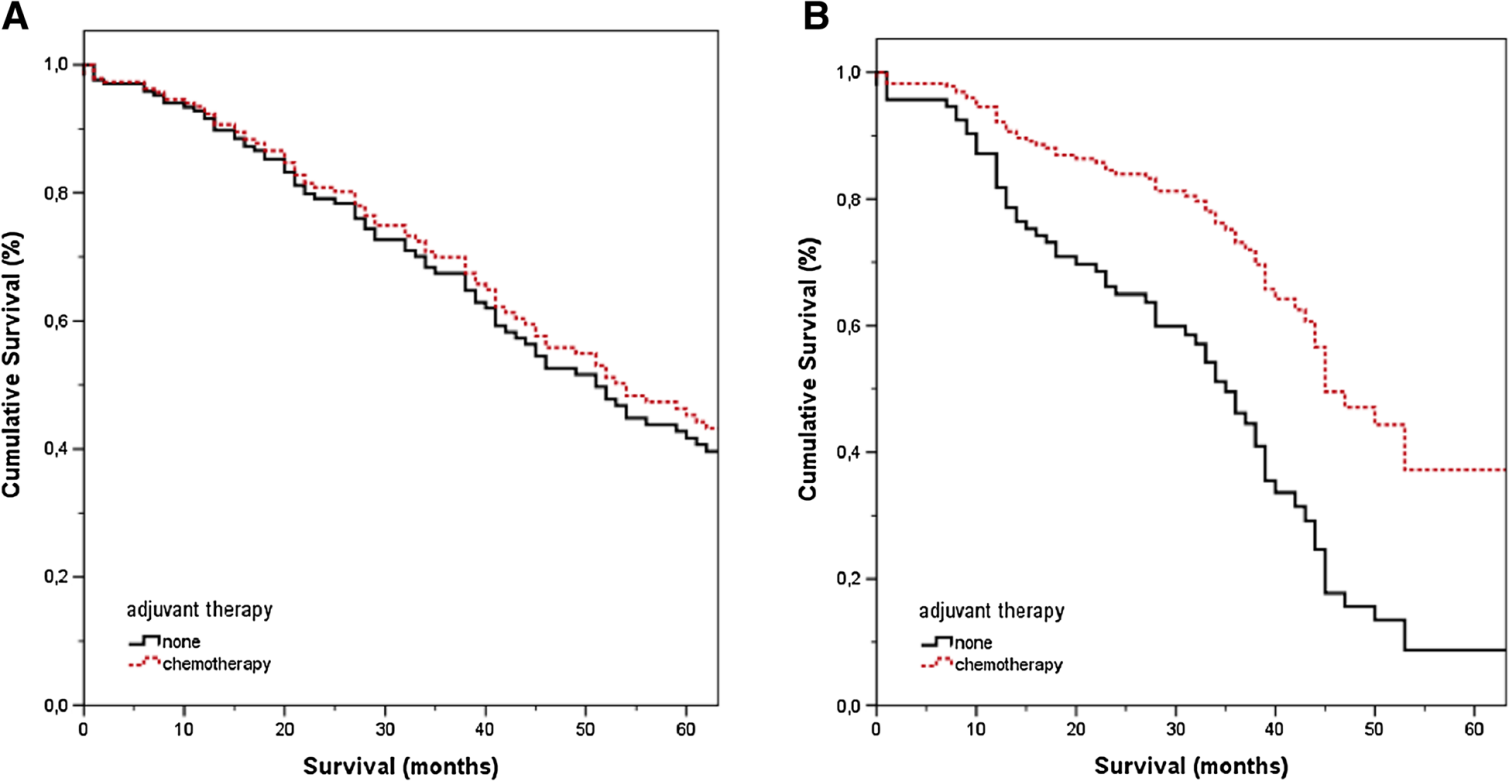
**Influence of adjuvant chemotherapy on overall survival after potentially curative resection of colorectal liver metastases depending on patients’ risk status. A**. Overall survival of patients with a MSKCC-CRS ≤ 2 stratified for the type of adjuvant therapy (p = 0.53). **B**. Overall survival of patients with a MSKCC-CRS > 2 stratified for the type of adjuvant therapy (p = 0.007). Data are presented as Cox proportional hazards.

Owing to the known prognostic value of the MSKCC-CRS in our patients as well as other patient cohorts [3,10,11], we stratified the multivariate model for a MSKCC-CRS of ≤ 2 and > 2 to further evaluate a potential benefit of adjuvant chemotherapy in low and high-risk patients, respectively (Table 4). The multivariate analysis of patients with a MSKCC-CRS ≤ 2 revealed no survival benefit of adjuvant chemotherapy (HR 0.90; 95% CI 0.57–1.43; p = 0.67). However, there was a strong advantage in overall survival for patients who received adjuvant chemotherapy in the multivariate model restricted to patients with a MSKCC-CRS > 2 (HR 0.40; 95% CI 0.23–0.70; p = 0.001) (Figure 1).

**Table 4:** Multivariate analysis of factors associated with overall survival in patients who underwent potentially curative resection for colorectal cancer liver metastases stratified for patients’ MSKCC clinical risk score

**Variable**	**Comparison**	**Hazard ratio**	**95% CI**	***P *** **Value**
**MSKCC clinical risk score ≤ 2**				
**Age**	-	1.01	0.99 – 1.04	0.20
**Distribution of metastasis**	Bilobar vs. unilobar	1.80	1.12 – 2.92	0.01
**Initial stage of disease (UICC)**				0.41
	1	Reference		
	2	1.96	0.80 – 4.81	0.13
	3	1.92	0.80 – 4.59	0.14
	4	1.52	0.60 – 3.83	0.36
**Adjuvant chemotherapy**	Adjuvant CTx vs. no adjuvant CTx	0.90	0.57 – 1.43	0.67
**MSKCC clinical risk score > 2**				
**Age**	-	1.00	0.98 – 1.03	0.61
**Distribution of metastasis**	Bilobar vs. unilobar	1.65	0.98 – 2.77	0.06
**Initial stage of disease (UICC)**				0.21
	1	Reference		
	2	0.21	0.02 – 2.27	0.20
	3	0.60	0.07 – 4.79	0.63
	4	0.38	0.05 – 3.03	0.36
**Adjuvant chemotherapy**	Adjuvant CTx vs. no adjuvant CTx	0.40	0.23 – 0.70	0.001

CTx, Chemotherapy.

**Figure 2:**
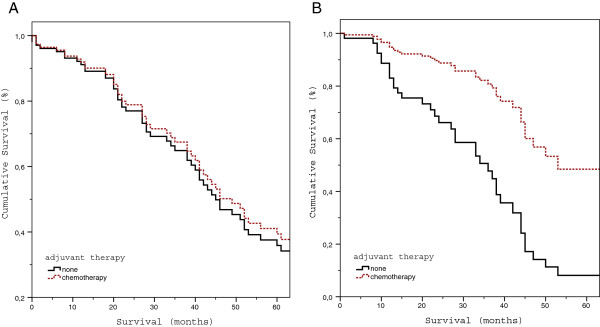
**Influence of adjuvant chemotherapy on overall survival after potentially curative resection of colorectal liver metastases in patients with a borderline risk status. A**. Overall survival of patients with a MSKCC-CRS 2 stratified for the type of adjuvant therapy (p = 0.62). **B**. Overall survival of patients with a MSKCC-CRS 3 stratified for the type of adjuvant therapy (p = 0.01). Data are presented as Cox proportional hazards.

We performed subgroup analyses to further elucidate the efficacy of adjuvant chemotherapy in patients with a borderline risk status and to assess the adequacy of the applied cut-off for the MSKCC-CRS (≤ 2 vs. > 2) to stratify patients in a low-and high-risk group. These analyses revealed that adjuvant chemotherapy failed to improve survival in patients with a MSKCC-CRS of 2, whereas it was associated with a significant survival benefit in patients with a MSKCC-CRS of 3 (Figure 2).

## Discussion

There is limited evidence on the efficacy of adjuvant therapy to prolong survival after potentially curative resection of CRC liver metastases. The available randomized controlled trials were not placebo-controlled and lacked sufficient statistical power to detect differences in survival [22-25]. Mitry et al. published a pooled analysis of two randomized trials that evaluated adjuvant therapy with bolus 5-FU/LV on a combined population of 302 patients [7]. In this study the benefit of adjuvant therapy for progression-free survival and overall survival failed to reach statistical significance. However, multivariate analyses adjusting for the number of metastases, prior chemotherapy (analysis of progression-free survival) and the disease-free interval (analysis of overall survival) favored adjuvant chemotherapy with regard to progression-free survival (HR 1.39; 95% CI 1.04–1.85) and overall survival (HR 1.39; 95% CI 1.00–1.93).

The benefit in overall survival reported by Mitry et al. for patients who received adjuvant therapy is very similar to the risk reduction observed in our study. While these authors did not evaluate the outcome after adjuvant therapy depending on patients’ preoperative risk status, the lack of a clear survival benefit with adjuvant chemotherapy may in part also be caused by the use of less active protocols. Chemotherapy with 5-FU/LV has been the standard adjuvant chemotherapy for patients with colorectal liver metastases. Various studies on systemic therapy of metastatic CRC have demonstrated improved efficacy of chemotherapy protocols including oxaliplatin [26-28] or irinotecan [29-31] to 5-FU/LV. Together with studies that proved significantly better long-term outcome of patients receiving modern combination chemotherapy for adjuvant treatment after resection of the colorectal primary [20,32] these data raised the question, of efficacy of adjuvant chemotherapy after resection of colorectal liver metastases can be further improved by addition of oxaliplatin or irinotecan to 5-FU/LV. In a recently published phase III trial Ychou et al. evaluated adjuvant therapy after surgery for colorectal liver metastases using the 5-FU/LV backbone with or without irinotecan [33]. This study included 306 patients and failed to demonstrate a significant advantage in disease-free survival for patients who received adjuvant FOLFIRI.

The lack of stratified analyses considering patients’ risk status might serve as a further explanation, why the available studies failed to demonstrate a clear survival benefit for patients treated with adjuvant chemotherapy. Using the MSKCC-CRS that has been validated in several studies [3,10,11], we here show that adjuvant therapy is highly active in high-risk patients, whereas it is not associated with prolonged survival in patients with low-risk disease features. Our results confirm the findings by Parks et. who reported the long-term outcomes after adjuvant therapy in a cohort of 792 patients with hepatic resection at two institutions between 1991 and 1998 [34]. Although these authors showed a survival benefit of adjuvant chemotherapy in particular for patients with a MSKCC-CRS of 4 and 5, one should note that in this analysis 5-FU based chemotherapy was administered only, without addition of oxaliplatin, irinotecan. Further evidence supporting the use of adjuvant therapy primarily in high-risk patients is provided by a recent multi-institutional study on 1471 patients who underwent resection for solitary, metachronous and primarily resectable metastases without extrahepatic disease [35]. In this study of patients with potentially curative resection modern chemotherapy protocols were applied. The authors reported a benefit of adjuvant chemotherapy in patients with a metastasis > 5 cm or more in diameter, whereas there was no influence of adjuvant chemotherapy in patients with tumors less a size than 5 cm. Together with the results of our study these data suggest that allocation in future randomized controlled trials should be stratified for patients’ risk status to identify those patients who benefit from adjuvant chemotherapy. The ideal tool to evaluate patients’ risk status, however, remains to be determined. Although the present study favors the MSKCC-CRS, there is evidence that the predictive value of this scoring system may be further improved by additional/alternative molecular or clinical markers [36]. Additional studies are required to determine the benefit of adjuvant chemotherapy based on the results of various risk assessment tools in order to identify the most accurate classification system.

The finding that adjuvant chemotherapy improves survival exclusively in patients with high-risk clinical features of disease supports efforts to identify prognostic biomarkers indicating patients with a high-likelihood of tumor relapse and cancer-related death. Numerous studies have so far investigated various cellular, molecular or genetic markers as predictors of outcome in patients with primary and metastatic CRC [37-39]. The inconsistent findings of most studies, which may be explained by insufficient statistical power, differences in the experimental setup and patient cohorts have prevented the widespread use of predictive markers in patients with primary and metastatic CRC. One should, however, note that there are far less data on predictors of poor long-term outcome for patients undergoing potentially curative resection of colorectal liver metastases. There is evidence that expression of certain markers within resected liver metastases may predict disease recurrence and survival [40-42]. In a recently published analyses on 107 patients who underwent potentially curative resection for colorectal liver metastases we demonstrated that preoperative level of circulating placental growth factor was associated independently with the risk of disease recurrence [43]. While these results need to be validated in independent patient populations, further studies are required to determine the optimal timing for the assessment of circulating biomarkers as predictors of outcome after resection of CRC liver metastases [44]. These data should present the basis for the conduction of prospective clinical trials evaluating the efficacy of adjuvant chemotherapy depending on patients’ angiogenic profile.

## Conclusion

In conclusion, the present study shows that adjuvant chemotherapy after potentially curative resection of CRC liver metastases is associated with favorable outcome in high-risk patients, whereas it offers no survival benefit in patients with low-risk features of disease. The MSKCC-CRS might thus offer a tool to tailor adjuvant therapy after resection of CRC liver metastases. Although validation of these results is required in independent patient cohorts, the present data strongly suggest that patients in studies on adjuvant chemotherapy after potentially curative resection of colorectal liver metastases should be stratified for their risk status.

## Competing interests

The authors declare that they have no competing interests.

## Authors’ contributions

This study was designed by NNR, CR and MK. The article was written by NNR and CR. NNR, CR, HSB, JW and MK were involved in data acquisition. NNR performed the statistical analyses. CR, HSB, DJ, MWB, JW and MK critically revised the manuscript. All authors have read and approved the manuscript.

## Pre-publication history

The pre-publication history for this paper can be accessed here:

http://www.biomedcentral.com/1471-2407/14/174/prepub

## Supplementary Material

Additional file 1: Table S1 Kind of administered adjuvant chemotherapy. Click here for file

## References

[B1] Scheele J Stangl R Altendorf-Hofmann A Hepatic metastases from colorectal carcinoma: impact of surgical resection on the natural history Br J Surg 1990 77 11 1241 1246 10.1002/bjs.1800771115 2253003

[B2] Fong Y Fortner J Sun RL Brennan MF Blumgart LH Clinical score for predicting recurrence after hepatic resection for metastatic colorectal cancer: analysis of 1001 consecutive cases Ann Surg 1999 230 3 309 318 discussion 318–321 10.1097/00000658-199909000-00004 10493478 PMC1420876

[B3] Reissfelder C Rahbari NN Koch M Ulrich A Pfeilschifter I Waltert A Muller SA Schemmer P Buchler MW Weitz J Validation of prognostic scoring systems for patients undergoing resection of colorectal cancer liver metastases Ann Surg Oncol 2009 16 12 3279 3288 10.1245/s10434-009-0654-7 19688403

[B4] Francini G Petrioli R Lorenzini L Mancini S Armenio S Tanzini G Marsili S Aquino A Marzocca G Civitelli S Folinic acid and 5-fluorouracil as adjuvant chemotherapy in colon cancer Gastroenterology 1994 106 4 899 906 8143995 10.1016/0016-5085(94)90748-x

[B5] O'Connell MJ Mailliard JA Kahn MJ Macdonald JS Haller DG Mayer RJ Wieand HS Controlled trial of fluorouracil and low-dose leucovorin given for 6 months as postoperative adjuvant therapy for colon cancer J Clin Oncol 1997 15 1 246 250 8996149 10.1200/JCO.1997.15.1.246

[B6] André T Boni C Navarro M Tabernero J Hickish T Topham C Bonetti A Clingan P Bridgewater J Rivera F de Gramont A Improved overall survival with oxaliplatin, fluorouracil, and leucovorin as adjuvant treatment in stage II or III colon cancer in the MOSAIC trial J Clin Oncol 2009 27 19 3109 3116 10.1200/JCO.2008.20.6771 19451431

[B7] Mitry E Fields AL Bleiberg H Labianca R Portier G Tu D Nitti D Torri V Elias D O'Callaghan C Langer B Martignoni G Bouché O Lazorthes F Van Cutsem E Bedenne L Moore MJ Rougier P Adjuvant chemotherapy after potentially curative resection of metastases from colorectal cancer: a pooled analysis of two randomized trials J Clin Oncol 2008 26 30 4906 4911 10.1200/JCO.2008.17.3781 18794541

[B8] Nordlinger B Sorbye H Glimelius B Poston GJ Schlag PM Rougier P Bechstein WO Primrose JN Walpole ET Finch-Jones M Jaeck D Mirza D Parks RW Collette L Praet M Bethe U Van Cutsem E Scheithauer W Gruenberger T Perioperative chemotherapy with FOLFOX4 and surgery versus surgery alone for resectable liver metastases from colorectal cancer (EORTC Intergroup trial 40983): a randomised controlled trial Lancet 2008 371 9617 1007 1016 10.1016/S0140-6736(08)60455-9 18358928 PMC2277487

[B9] Nordlinger B Sorbye H Glimelius B Poston GJ Schlag PM Rougier P Bechstein WO Primrose JN Walpole ET Finch-Jones M Jaeck D Mirza D Parks RW Collette L Praet M Bethe U Van Cutsem E Scheithauer W Gruenberger T **Perioperative FOLFOX4 chemotherapy and surgery versus surgery alone for resectable liver metastases from colorectal cancer (EORTC 40983): long-term results of a randomised, controlled, phase 3 trial** Lancet Oncol 2013 14 12 1208 1215 10.1016/S1470-2045(13)70447-9 24120480

[B10] Merkel S Bialecki D Meyer T Muller V Papadopoulos T Hohenberger W Comparison of clinical risk scores predicting prognosis after resection of colorectal liver metastases J Surg Oncol 2009 100 5 349 357 10.1002/jso.21346 19572329

[B11] Zakaria S Donohue JH Que FG Farnell MB Schleck CD Ilstrup DM Nagorney DM Hepatic resection for colorectal metastases: value for risk scoring systems? Ann Surg 2007 246 2 183 191 10.1097/SLA.0b013e3180603039 17667495 PMC1933577

[B12] Tomlinson JS Jarnagin WR DeMatteo RP Fong Y Kornprat P Gonen M Kemeny N Brennan MF Blumgart LH D'Angelica M Actual 10-year survival after resection of colorectal liver metastases defines cure J Clin Oncol 2007 25 29 4575 4580 10.1200/JCO.2007.11.0833 17925551

[B13] Sobin LH Gospodarowicz MK Wittekind C UICC: TNM classification of malignant tumours 2009 7 New York: Wiley & Sons

[B14] Rahbari NN Zimmermann JB Koch M Bruckner T Schmidt T Elbers H Reissfelder C Weigand MA Buchler MW Weitz J IVC CLAMP: infrahepatic inferior vena cava clamping during hepatectomy–a randomised controlled trial in an interdisciplinary setting Trials 2009 10 94 10.1186/1745-6215-10-94 19825186 PMC2770522

[B15] Reissfelder C Rahbari NN Koch M Kofler B Sutedja N Elbers H Buchler MW Weitz J Postoperative course and clinical significance of biochemical blood tests following hepatic resection Br J Surg 2011 98 6 836 844 10.1002/bjs.7459 21456090

[B16] Rahbari NN Reissfelder C Koch M Elbers H Striebel F Buchler MW Weitz J The predictive value of postoperative clinical risk scores for outcome after hepatic resection: a validation analysis in 807 patients Ann Surg Oncol 2011 18 13 3640 3649 10.1245/s10434-011-1829-6 21674269

[B17] Rahbari NN Koch M Schmidt T Motschall E Bruckner T Weidmann K Mehrabi A Buchler MW Weitz J Meta-analysis of the clamp-crushing technique for transection of the parenchyma in elective hepatic resection: back to where we started? Ann Surg Oncol 2009 16 3 630 639 10.1245/s10434-008-0266-7 19130140

[B18] Rahbari NN Wente MN Schemmer P Diener MK Hoffmann K Motschall E Schmidt J Weitz J Buchler MW Systematic review and meta-analysis of the effect of portal triad clamping on outcome after hepatic resection Br J Surg 2008 95 4 424 432 10.1002/bjs.6141 18314921

[B19] Weh HJ Wilke HJ Dierlamm J Klaassen U Siegmund R Illiger HJ Schalhorn A Kreuser ED Hilgenfeld U Steinke B Weekly therapy with folinic acid (FA) and high-dose 5-fluorouracil (5-FU) 24-hour infusion in pretreated patients with metastatic colorectal carcinoma. A multicenter study by the Association of Medical Oncology of the German Cancer Society (AIO) Ann Oncol 1994 5 3 233 237 8186170 10.1093/oxfordjournals.annonc.a058799

[B20] André T Boni C Mounedji-Boudiaf L Navarro M Tabernero J Hickish T Topham C Zaninelli M Clingan P Bridgewater J Tabah-Fisch I de Gramont A Oxaliplatin, fluorouracil, and leucovorin as adjuvant treatment for colon cancer N Engl J Med 2004 350 23 2343 2351 10.1056/NEJMoa032709 15175436

[B21] André T Louvet C Maindrault-Goebel F Couteau C Mabro M Lotz JP Gilles-Amar V Krulik M Carola E Izrael V de Gramont A CPT-11 (irinotecan) addition to bimonthly, high-dose leucovorin and bolus and continuous-infusion 5-fluorouracil (FOLFIRI) for pretreated metastatic colorectal cancer. GERCOR Eur J Cancer 1999 35 9 1343 1347 10.1016/S0959-8049(99)00150-1 10658525

[B22] O'Connell MJ Adson MA Schutt AJ Rubin J Moertel CG Ilstrup DM Clinical trial of adjuvant chemotherapy after surgical resection of colorectal cancer metastatic to the liver Mayo Clin Proc 1985 60 8 517 520 10.1016/S0025-6196(12)60567-9 3894814

[B23] Mitry E Fields AL Bleiberg H Labianca R Portier G Tu D Nitti D Torri V Elias D O'Callaghan C Langer B Martignoni G Bouché O Lazorthes F Van Cutsem E Bedenne L Moore MJ Rougier Fluorouracil (FU) plus l-leucovorin (l-LV) versus observation after potentially curative resection of liver or lung metastases from colorectal cancer (CRC): results of the ENG (EORTC/NCIC CTG/GIVIO) randomized trial Proc Amer Soc Clin Oncol 2002 21 149a 592

[B24] Lopez-Ladron A Salvador J Bernabe R Observation versus postoperative chemotherapy after resection of liver metastases in patients with advanced colorectal cancer Proc Amer Soc Clin Oncol 2003 22 373 1497

[B25] Portier G Elias D Bouche O Rougier P Bosset JF Saric J Belghiti J Piedbois P Guimbaud R Nordlinger B Bugat R Lazorthes F Bedenne L Multicenter randomized trial of adjuvant fluorouracil and folinic acid compared with surgery alone after resection of colorectal liver metastases: FFCD ACHBTH AURC 9002 trial J Clin Oncol 2006 24 31 4976 4982 10.1200/JCO.2006.06.8353 17075115

[B26] André T Bensmaine MA Louvet C François E Lucas V Desseigne F Beerblock K Bouché O Carola E Merrouche Y Morvan F Dupont-André G de Gramont A Multicenter phase II study of bimonthly high-dose leucovorin, fluorouracil infusion, and oxaliplatin for metastatic colorectal cancer resistant to the same leucovorin and fluorouracil regimen J Clin Oncol 1999 17 11 3560 3568 10550155 10.1200/JCO.1999.17.11.3560

[B27] Giacchetti S Perpoint B Zidani R Le Bail N Faggiuolo R Focan C Chollet P Llory JF Letourneau Y Coudert B Bertheaut-Cvitkovic F Larregain-Fournier D Le Rol A Walter S Adam R Misset JL Lévi F Phase III multicenter randomized trial of oxaliplatin added to chronomodulated fluorouracil-leucovorin as first-line treatment of metastatic colorectal cancer J Clin Oncol 2000 18 1 136 147 10623704 10.1200/JCO.2000.18.1.136

[B28] Goldberg RM Sargent DJ Morton RF Fuchs CS Ramanathan RK Williamson SK Findlay BP Pitot HC Alberts SR A randomized controlled trial of fluorouracil plus leucovorin, irinotecan, and oxaliplatin combinations in patients with previously untreated metastatic colorectal cancer J Clin Oncol 2004 22 1 23 30 14665611 10.1200/JCO.2004.09.046

[B29] Rougier P Van Cutsem E Bajetta E Niederle N Possinger K Labianca R Navarro M Morant R Bleiberg H Wils J Awad L Herait P Jacques C Randomised trial of irinotecan versus fluorouracil by continuous infusion after fluorouracil failure in patients with metastatic colorectal cancer Lancet 1998 352 9138 1407 1412 10.1016/S0140-6736(98)03085-2 9807986

[B30] Köhne CH van Cutsem E Wils J Bokemeyer C El-Serafi M Lutz MP Lorenz M Reichardt P Ruckle-Lanz H Frickhofen N Fuchs R Mergenthaler HG Langenbuch T Vanhoefer U Rougier P Voigtmann R Müller L Genicot B Anak O Nordlinger B Phase III study of weekly high-dose infusional fluorouracil plus folinic acid with or without irinotecan in patients with metastatic colorectal cancer: European organisation for research and treatment of cancer gastrointestinal group study 40986 J Clin Oncol 2005 23 22 4856 4865 10.1200/JCO.2005.05.546 15939923

[B31] Saltz LB Cox JV Blanke C Rosen LS Fehrenbacher L Moore MJ Maroun JA Ackland SP Locker PK Pirotta N Elfring GL Miller LL Irinotecan plus fluorouracil and leucovorin for metastatic colorectal cancer. Irinotecan Study Group N Engl J Med 2000 343 13 905 914 10.1056/NEJM200009283431302 11006366

[B32] Haller DG Tabernero J Maroun J de Braud F Price T Van Cutsem E Hill M Gilberg F Rittweger K Schmoll HJ Capecitabine plus oxaliplatin compared with fluorouracil and folinic acid as adjuvant therapy for stage III colon cancer J Clin Oncol 2011 29 11 1465 1471 10.1200/JCO.2010.33.6297 21383294

[B33] Ychou M Hohenberger W Thezenas S Navarro M Maurel J Bokemeyer C Shacham-Shmueli E Rivera F Kwok-Keung Choi C Santoro A A randomized phase III study comparing adjuvant 5-fluorouracil/folinic acid with FOLFIRI in patients following complete resection of liver metastases from colorectal cancer Ann Oncol 2009 20 12 1964 1970 10.1093/annonc/mdp236 19567451

[B34] Parks R Gonen M Kemeny N Jarnagin W D'Angelica M DeMatteo R Garden OJ Blumgart LH Fong Y Adjuvant chemotherapy improves survival after resection of hepatic colorectal metastases: analysis of data from two continents J Am Coll Surg 2007 204 5 753 761 discussion 761–753 10.1016/j.jamcollsurg.2006.12.036 17481478

[B35] Adam R Bhangui P Poston G Mirza D Nuzzo G Barroso E Ijzermans J Hubert C Ruers T Capussotti L Ouellet JF Laurent C Cugat E Colombo PE Milicevic M Is perioperative chemotherapy useful for solitary, metachronous, colorectal liver metastases? Ann Surg 2010 252 5 774 787 10.1097/SLA.0b013e3181fcf3e3 21037433

[B36] Maithel SK Gonen M Ito H Dematteo RP Allen PJ Fong Y Blumgart LH Jarnagin WR D'Angelica MI Improving the clinical risk score: an analysis of molecular biomarkers in the era of modern chemotherapy for resectable hepatic colorectal cancer metastases Surgery 2012 151 2 162 170 10.1016/j.surg.2011.07.020 21982065

[B37] Fritzmann J Morkel M Besser D Budczies J Kosel F Brembeck FH Stein U Fichtner I Schlag PM Birchmeier W A colorectal cancer expression profile that includes transforming growth factor beta inhibitor BAMBI predicts metastatic potential Gastroenterology 2009 137 1 165 175 10.1053/j.gastro.2009.03.041 19328798

[B38] Rahbari NN Aigner M Thorlund K Mollberg N Motschall E Jensen K Diener MK Buchler MW Koch M Weitz J Meta-analysis shows that detection of circulating tumor cells indicates poor prognosis in patients with colorectal cancer Gastroenterology 2010 138 5 1714 1726 10.1053/j.gastro.2010.01.008 20100481

[B39] Kahlert C Klupp F Brand K Lasitschka F Diederichs S Kirchberg J Rahbari N Dutta S Bork U Fritzmann J Reissfelder C Koch M Weitz J Invasion front-specific expression and prognostic significance of microRNA in colorectal liver metastases Cancer Sci 2011 102 10 1799 1807 10.1111/j.1349-7006.2011.02023.x 21722265

[B40] Nash GM Gimbel M Shia J Nathanson DR Ndubuisi MI Zeng ZS Kemeny N Paty PB KRAS mutation correlates with accelerated metastatic progression in patients with colorectal liver metastases Ann Surg Oncol 2010 17 2 572 578 10.1245/s10434-009-0605-3 19727962

[B41] Yopp AC Shia J Butte JM Allen PJ Fong Y Jarnagin WR Dematteo RP D'Angelica MI CXCR4 Expression predicts patient outcome and recurrence patterns after hepatic resection for colorectal liver metastases Ann Surg Oncol 2011 19 Suppl 3 S339 S346 21584832 10.1245/s10434-011-1774-4

[B42] Rahbari NN Bork U Motschall E Thorlund K Buchler MW Koch M Weitz J Molecular detection of tumor cells in regional lymph nodes is associated with disease recurrence and poor survival in node-negative colorectal cancer: a systematic review and meta-analysis J Clin Oncol 2012 30 1 60 70 10.1200/JCO.2011.36.9504 22124103

[B43] Rahbari NN Reissfelder C Muhlbayer M Weidmann K Kahlert C Buchler MW Weitz J Koch M Correlation of circulating angiogenic factors with circulating tumor cells and disease recurrence in patients undergoing curative resection for colorectal liver metastases Ann Surg Oncol 2011 18 8 2182 2191 10.1245/s10434-011-1761-9 21598056

[B44] Yoon SS Kim SH Gonen M Heffernan NM Detwiller KY Jarnagin WR D'Angelica M Blumgart LH Tanabe KK Dematteo RP Profile of plasma angiogenic factors before and after hepatectomy for colorectal cancer liver metastases Ann Surg Oncol 2006 13 3 353 362 10.1245/ASO.2006.03.060 16474912

