# Supplementary material for: Adjuvant therapy after resection of colorectal liver metastases: the predictive value of the MSKCC clinical risk score in the era of modern chemotherapy
Source: BMC Cancer. 2014 Mar 11;14:174. doi: 10.1186/1471-2407-14-174 (PMC4008001; doi:10.1186/1471-2407-14-174)
Supplement: Additional file 1: Table S1 — Kind of administered adjuvant chemotherapy. [file 1471-2407-14-174-S1.doc]

**Supplemental Table 1: Kind of administered adjuvant chemotherapy**

|  | **n** | **MSKCC clinical risk score** | |
| --- | --- | --- | --- |
|  |  | ≤ 2 (%) | > 2 (%) |
| No adjuvant chemotherapy | 181 (61) | 111 (62) | 70 (38) |
| Adjuvant chemotherapy | 116 (39) | 59 (51) | 57 (49) |
| 5-FU/Leucovorin | 38 (28) | 18 (47) | 20 (53) |
| FOLFOX4 | 62 (46) | 37 (60) | 25 (40) |
| FOLFIRI | 16 (12) | 4 (25) | 12 (75) |
